# Biases in Prokaryotic Community Amplicon Sequencing Affected by DNA Extraction Methods in Both Saline and Non-saline Soil

**DOI:** 10.3389/fmicb.2018.01796

**Published:** 2018-08-03

**Authors:** Kehui Xie, Yong Deng, Xuze Zhang, Xueting Wang, Guangbo Kang, Liang Bai, He Huang

**Affiliations:** ^1^Department of Biochemical Engineering, School of Chemical Engineering and Technology, Tianjin University, Tianjin, China; ^2^Key Laboratory of Systems Bioengineering, Ministry of Education, Tianjin University, Tianjin, China; ^3^School of Chemistry and Chemical Engineering, Qinghai University for Nationalities, Xining, China

**Keywords:** DNA extraction, physical lysis, soil microbiome, microbial diversity, microbial community

## Abstract

High-throughput amplicon sequencing technology has been widely used in soil microbiome studies. Here, we estimated the bias of amplicon sequencing data affected by DNA extraction methods in a saline soil, and a non-saline normal soil was used as a control. Compared with the normal soil, several unique points were observed in the saline soil. The soil washing pretreatment can improve not only DNA quantity and quality but also microbial diversities in the saline soil; therefore, we recommend the soil washing pretreatment for saline soils especially hypersaline soils that cannot be achieved with detectable DNA amounts without the pretreatment. Also, evenness indices were more easily affected by DNA extraction methods than richness indices in the saline soil. Moreover, proportions of Gram-positive bacteria had significant positive correlations with the achieved microbial diversities within replicates of the saline soil. Though DNA extraction methods can bias the microbial diversity or community and relative abundances of some phyla/classes can vary by a factor of more than five, soil types were still the most important factor of the whole community. We confirmed good comparability in the whole community, but more attention should be paid when concentrating on an exact diversity value or the exact relative abundance of a certain taxon. Our study can provide references for the DNA extraction from saline and non-saline soils and comparing sequencing data across studies who may employ different DNA extraction methods.

## Introduction

The fast-growing high-throughput sequencing technology has considerably changed our understanding of microbial communities in all kinds of environments on Earth (Bates et al., 2011; Caporaso et al., 2011a,b). Compared with shotgun metagenomics, the amplicon sequencing was much more cost-effective and more widely used (Caporaso et al., 2011b), but additional PCR amplification may bias microbiome studies (Gohl et al., 2016). Other procedures, such as DNA extraction, library preparation, and downstream bioinformatic analysis, may also cause biases (Wust et al., 2016; Sinha et al., 2017; Zielinska et al., 2017). Two recent reports have confirmed that DNA extraction had more effects than other procedures in human fecal microbiome studies (Costea et al., 2017; Sinha et al., 2017).

Lots of microorganisms distributed in extremely complex and diverse soil communities (Daniel, 2005; Bardgett and van der Putten, 2014). Humus, contaminants, salts, and many other substances made DNA extraction a crucial and challenging procedure in soil metagenomic studies (Steffan et al., 1988; Tebbe and Vahjen, 1993). Soil DNA extraction methods can be divided into direct methods and indirect methods (Daniel, 2005) with the former being more widely used (Zhou et al., 1996; Robe et al., 2003; Philippot et al., 2010). Moreover, DNA solutions achieved with manual methods were often contaminated by the humus, which would interfere with further PCR amplification and sequencing (Zhou et al., 1996; Arbeli and Fuentes, 2007). Therefore, DNA purification steps were also needed for crude DNA from manual methods.

Previous reports have estimated the biases associated with different DNA extraction methods in various kinds of soil or sediment microbiomes (Purohit and Singh, 2009; Plassart et al., 2012; Cruaud et al., 2014; Natarajan et al., 2016; Wust et al., 2016; Gupta et al., 2017; Santos et al., 2017; Zielinska et al., 2017). Few studies have evaluated the bias in saline soil or saline sediment microbiomes (Carrigg et al., 2007; Purohit and Singh, 2009; Siddhapura et al., 2010; Natarajan et al., 2016). Saline or hypersaline ecosystems, such as salt lakes, playas, and salt salterns, are globally distributed (Oren, 2002; Ventosa et al., 2008). Lots of salt and low biomass often exist in soils or sediments from these ecosystems (Keshri et al., 2013; Xie et al., 2017). Enough DNA can be difficult to achieve from saline soils or saline sediments (Natarajan et al., 2016; Xie et al., 2017). Therefore, more studies need doing to optimize DNA extraction methods for saline soils or saline sediments.

The widely used DNA extraction methods, which also included many commercial kits, usually contained bead-beating lysis steps before further DNA extraction (Philippot et al., 2010; Natarajan et al., 2016). Previous studies (Leff et al., 1995; Kauffmann et al., 2004; Lakay et al., 2007; Purohit and Singh, 2009) have reported that higher DNA yield and quality were achieved with the bead beating step than other physical steps, such as freezing and thawing, microwave heating, and liquid nitrogen grinding. However, some studies have confirmed that high DNA yield or quality did not correspond to high microbial diversities (Cruaud et al., 2014; Zielinska et al., 2017). Several recent reports (Natarajan et al., 2016; Wust et al., 2016; Zielinska et al., 2017) only employed methods including the bead-beating step, ignoring other physical lysis steps when estimating the biases of high-throughput amplicon sequencing data. The microbial diversity and community biases caused only by different physical lysis methods need to be clarified.

In the present study, we estimated biases associated with different DNA extraction methods in saline and non-saline soil microbiomes, and microbiome biases associated only with different physical lysis steps were also estimated. The estimated amplicon sequencing biases include raw data, qualified data, alpha diversities, beta diversities, microbial community compositions, phylogenetic analyses, and predictive functional compositions. The quality and quantity of achieved DNA were estimated, too. Based on these estimates, we also made several suggestions for soil DNA extraction methods.

## Materials and Methods

### Soil Sample Collection

Two saline soil samples (SS1, SS2; **Table 1**) were collected in spring 2017 at a depth of 0–10 cm in Binhai New Area, a coastal area in Tianjin, China. They were typical thalassohaline soils. The non-saline normal soil was collected in summer 2016 at a depth of 0–10 cm in Water Park, Tianjin. The sampling method was same as before (Xie et al., 2017): subsamples at four vertices of a one-meter square were mixed together into a representative sample. Roots, plants debris, and stones were removed from soils. Sampling locations and altitudes were recorded with a GPS locator (**Table 1**). Collected soils were stored into sterile plastic bags and transported to the laboratory in an ice box. For each soil sample, a part of soil was stored at 4°C for physical and chemical analyses, the others were stored at -80°C for the DNA extraction.

**Table 1:** Soil physical, chemical, and geographical properties.

Properties	NS	SS1	SS2
pH	8.9	8.6	8.8
TOC (g/kg)	22.2	8.4	ND
TN (g/kg)	2.4	2.5	ND
K (g/kg)	17.2	16.3	ND
P (g/kg)	0.9	0.4	ND
WC (%)	16.1	8.4	14.2
EC (dS/m)	0.32	16.39	34.9
Locations	39°05′15″N,	38°48′06″N,	38°48′14″N,
	117°10′23″E	117°30′35″E	117°30′32″E
Altitude (m)	4	3	3

*ND, not detected; NS, normal soil; SS, saline soil; TOC, total organic carbon; TN, total nitrogen; WC, water contents; EC, electrical conductivity.*

### Physical and Chemical Determinations

Before physical and chemical determinations, soils were air-dried and filtered through a 2-mm sieve. Soil pH and electrical conductivity (EC) were measured in slurries with soil/water (w/w) ratio 1:2.5 and 1:5, respectively. Water contents were determined by drying fresh soils at 105°C to a constant mass. Total organic carbon (TOC) was detected with the potassium dichromate heating oxidation method (Schumacher, 2002). The Kjeldahl method was used to detect total nitrogen (TN) contents. Phosphorus and potassium contents were determined with an inductively coupled plasma optical emission spectrometry (700 series; Agilent technologies, United States). All measures were conducted in duplicate, then took the means (**Table 1**).

### DNA Extraction

DNA extraction methods in the present study were summarized in **Table 2**. Zhou’s method (Zhou et al., 1996) and ISO 11063 method (Martin-Laurent et al., 2001; Philippot et al., 2010) were two commonly used manual DNA extraction methods from soils. We also selected the modified Zhou’s method (Natarajan et al., 2016), because it was modified for extracting DNA from seafloor sediments and the sediments were saline same as thalassohaline soils in the present study.

**Table 2:** Summary of DNA extraction methods in the present study.

Methods	Physical lysis steps	Extraction buffers	Enzyme lysis reagents	Purification chemicals
ZL	Liquid nitrogen grinding	100 mM Tris-HCl, 100 mM EDTA, 100 mM sodium phosphate, 1.5 M NaCl, 1% CTAB, PH = 8.0	SDS, Protease K	Chloroform, isoamyl alcohol, isopropanol
ZF	Freezing and Thawing	100 mM Tris-HCl, 100 mM EDTA, 100 mM sodium phosphate, 1.5 M NaCl, 1% CTAB, PH = 8.0	SDS, Protease K	Chloroform, isoamyl alcohol, isopropanol
ZB	Bead Beating	100 mM Tris-HCl, 100 mM EDTA, 100 mM sodium phosphate, 1.5 M NaCl, 1% CTAB, PH = 8.0	SDS, Protease K	Chloroform, isoamyl alcohol, isopropanol
MB	Bead Beating	100 mM Tris-HCl, 100 mM EDTA, 100 mM sodium phosphate, 1.5 M NaCl, 1% CTAB, PH = 8.0	SDS, Protease K, Lysozyme	PCI, isopropanol, sodium acetate
IB	Bead Beating	100 mM Tris-HCl, 100 mM EDTA, 100 mM NaCl, 1% PVP40, 2% SDS, PH = 8.0	SDS	Sodium acetate, isopropanol

*ZL, ZF represented Zhou’s method with liquid nitrogen grinding, freezing and thawing, respectively. ZB, MB, and IB represented Zhou’s method (Zhou et al., 1996), the modified Zhou’s method (Natarajan et al., 2016), and ISO 11063 method (Martin-Laurent et al., 2001; Philippot et al., 2010) with the same bead beating step, respectively.*

We designed experiments according to our purposes: (a) different physical lysis steps (liquid nitrogen grinding, freezing and thawing, and bead beating) following the same method (Zhou’s method in the present study); (b) the same physical lysis step (bead beating) following different methods (**Table 2**). The original Zhou’s method (Zhou et al., 1996) was used for a large amount of soil (5 g); we scaled down the original soil weight to 0.3 g and corresponding solutions to fit into 2 ml centrifuge tubes. The liquid nitrogen grinding step was applied by grinding fresh soil (about 0.5 g) in liquid nitrogen with a mortar and a pestle for 5 min. The mortar and pestle were firstly washed with 75% ethanol, then sterilized at 121°C for 20 min in an autoclave. The freezing and thawing step was three cycles of freezing at -80°C for 10 min and thawing at 65°C for 10 min after mixing soil with the extraction buffer. The bead beating step was applied by mixing soil with equal weight of 0.4–0.6 mm-diameter glass beads and two 4 mm-diameter glass beads; then add the extraction buffer, vortex blend at 2800 rpm for 5 min, and homogenize in a tissuelyser (Tissuelyser II; QIAGEN, Germany) at 30 Hz for 30 s for three cycles. The bead beating step was the same across different following methods. Moreover, we used PowerSoil commercial kit (Mo Bio Laboratories, Carlsbad, CA, United States) following manufacturer’s instructions with the alternative protocol for low-biomass soils as a control. Each extraction method was conducted in triplicate for both the saline soil and the normal soil.

To overcome salt interferences, we tested effects of the soil washing pretreatment with phosphate-buffered saline (PBS) in saline soils: washing 3 g saline soil with 30 ml PBS in a 50 ml centrifuge tube, gently vortex blending for 15 min, and centrifuging at 8000 *g* for 10 min. For hypersaline soils, the amount of PBS can be increased. Then the PowerSoil kit was used for further DNA extraction. Except the saline soil SS1 for further sequencing, we also tested another saline soil SS2 with much higher salinity (**Table 1**). The quality and quantity of DNA were considerably improved after soil washing (Supplementary Table S2).

The soil after washing froze so quickly in liquid nitrogen that the grinding cannot be applied. Therefore, except Zhou’s method with liquid nitrogen grinding step (ZL), the saline soil was all pretreated with PBS washing within other methods in the present study.

All crude DNA solutions achieved with manual methods were further purified with PowerClean DNA clean-up kit (Mo Bio Laboratories, Carlsbad, CA, United States); DNA solutions achieved with PowerSoil kit needed no further purification. Then, quality and quantity of purified DNA were measuring with an ultraviolet (UV) spectrophotometer (Q5000; Quawell, United States); DNA fragment sizes were measured by the electrophoresis on 1% agarose gels.

### Analysis of Amplicon Sequencing Data

The V4 regions of prokaryotic 16S rRNA genes were amplified with primers 515F and 806R (Bates et al., 2011; Caporaso et al., 2011b) in triplicate. The primers were fused with a barcode and an Illumina adaptor. Triplicate PCR products were pooled and sequenced in the Illumina HiSeq2500 platform (Caporaso et al., 2012) by Novogene (Beijing, China), generating 250 bp paired-end reads.

Generated reads were demultiplexed based on the barcode of each sample, and barcodes and primers were removed from reads. Raw tags were then generated by merging paired-end reads of each sample with FLASH software (Magoč and Salzberg, 2011). Raw tags were qualified with the script *split_libraries_fastq.py* in QIIME 1.9.1 (Caporaso et al., 2010b): (a) truncate at the first base call when existing three or more consecutive low-quality base calls with Phred quality scores lower than 20; (b) remove tags with a low percentage of consecutive high-quality base calls (lower than 75%). We performed both *de novo* and reference-based chimera detections with the script *identify_chimeric_seqs.py* in QIIME (Caporaso et al., 2010b) against the RDP database (Cole et al., 2007) through UCHIME algorithm (Edgar et al., 2011). Effective tags were obtained. Then, effective tags were clustered together to OTUs with ≥97% similarity by UPARSE algorithm (Edgar, 2013). The most abundant sequence in each OTU was picked out as a representative. Moreover, all representative sequences were assigned with RDP classifier (Wang et al., 2007) against the Greengenes database (DeSantis et al., 2006) in QIIME. A phylogenetic tree, which was used for further UniFrac distance (Lozupone and Knight, 2005) calculation, was constructed with FasTree (Price et al., 2012) after aligning against the Greengenes core set with PyNAST (Caporaso et al., 2010a) in QIIME.

### Prokaryotic Diversity and Functional Prediction

Further alpha and beta diversity analyses were conducted after normalizing all samples with 64500 sequences per sample. For alpha diversity indices, richness indices (observed OTUs, Chao1 estimators), Pielou evenness index, Shannon and Simpson diversity indices, and Good’s coverage values were calculated in QIIME. For the beta diversity, principal coordinate analysis (PCoA) was conducted with the weighted Unifrac distance in QIIME. Venn diagrams were drawn with VennDiagram package in R (3.31) and OTUs that had total sequences in each group more than two were included. Heat map was conducted with Pheatmap package in R. PerMANOVA was conducted with PAST software (version 3.16) (Hammer et al., 2001), which was based on the Bray-Curtis distance at the OTU level. The linear discriminant analysis (LDA) effect size (LEfSe) (Segata et al., 2011) was carried out to discover biomarker taxa in each method with the threshold LDA value of two (**Figure 4**).

The mean nearest taxon distance (MNTD) was used to estimate phylogenetic clustering extents of taxa in a sample, and the net relatedness index (NTI) was standardized MNTD measures of taxa in a sample (Webb et al., 2002). Low MNTD and high NTI values indicate strong phylogenetic clustering extent of taxa (Webb et al., 2002). MNTD was calculated based on an OTU table and a corresponding phylogenetic tree with Picante (Kembel et al., 2014) package in R. The OTU table was generated through UPARSE algorithm (Edgar, 2013) with ≥15 sequences in each OTU.

The closed-reference biom table was generated with the script *pick_closed_reference_otus.py* in QIIME against the Greengenes database (version 13-5) (DeSantis et al., 2006) for PICRUSt (Langille et al., 2013) functional predictions. We conducted the prediction based on the KEGG Orthology database (Kanehisa et al., 2012). The weighted nearest sequenced taxon index (NSTI) (Langille et al., 2013) was also calculated for characterizing the predictive accuracy: increasing NSTI values means decreasing accuracies. The closed-reference biom table was also used for predictions of Gram-positive bacteria proportions in soil microbiomes using BugBase software (Ward et al., 2017) with default parameters.

### Data Availability

Well-assembled raw tags were stored at NCBI SRA (Sequence Read Archive) database with the BioProject accession number SRP125719. BioSample accession numbers of all replicate were listed in Supplementary Table S3.

## Results

### Soil Physical and Chemical Parameters

The saline soil differed greatly from the normal soil in physical and chemical parameters, especially electrical conductivity (**Table 1**). The salinity of two saline soil samples (EC > 16 dS/m) was much higher than that of the non-saline normal soil (EC = 0.32 dS/m). Except salinity, the saline soil SS1 used for further sequencing had similar pH with the normal soil NS, but TOC and WC were much lower in the saline soil than the normal soil (**Table 1**).

### DNA Quality and Quantity

The sizes of most DNA fragments after purification were more than 15 kb, and the DNA band intensity of PowerSoil kit was clearly higher than other methods (Supplementary Figure S1). Significantly (*P* < 0.01, Wilcoxon signed) higher DNA quantity was also observed in PowerSoil kit than other five manual methods in both normal soil and saline soil (Supplementary Table S1). Compared with manual methods, PowerSoil kit achieved DNA with significantly higher OD_260_/OD_280_ ratios (*P* = 0.017, Mann–Whitney) and significantly lower OD_260_/OD_230_ ratios (*P* = 0.032, Mann–Whitney) in the normal soil. The same trend was also observed in the saline soil, though it was unsignificant for OD_260_/OD_280_ (*P* = 0.36). Moreover, both electrophoresis and significant test (*P* = 0.006, Wilcoxon signed) confirmed higher DNA quantity achieved from the normal soil than the saline soil, but we observed no significant DNA quantity difference among five manual methods in both saline soil (*P* = 0.089, Kruskal–Wallis) and normal soil (*P* = 0.217). While variations of DNA quantity or quality existed, the purified DNA from all methods can be PCR-amplified and further sequenced.

### Statistics of Sequencing Data

Total raw tags from all samples was 3,147,037, of which 1,561,962 tags belonged to the normal soil with a mean of 86775 ± 6854 (s.d.) and 1,585,075 tags to the saline soil with a mean of 88060 ± 6615 (Supplementary Table S3). We observed no significant difference of raw tag numbers between the normal soil and the saline soil (*P* = 0.522, Wilcoxon signed) or among six extraction methods in both the normal soil (*P* = 0.183, Kruskal–Wallis) and the saline soil (*P* = 0.622, Kruskal–Wallis). We have filtered out 274,156 low-quality tags or chimeras, generating overall 2,872,881 effective tags. 1,405,136 of these effective tags belonged to the normal soil with a mean of 78063 ± 6560 (s.d.), other effective tags belonged to the saline soil with a mean of 81541 ± 7148 (Supplementary Table S3). Effective tags were also unsignificantly different between the two soils (*P* = 0.067, Wilcoxon signed) or among six extraction methods in both the normal soil (*P* = 0.177, Kruskal–Wallis) and the saline soil (*P* = 0.648, Kruskal–Wallis). Effective ratios (effective tags/raw tags) of two samples were also summarized (Supplementary Table S3). Significantly (*P* = 0.001, Wilcoxon signed) higher effective ratios were observed in the saline soil (0.93 ± 0.022) than the normal soil (0.90 ± 0.036), but no significant difference was observed among six different methods in both the saline soil (*P* = 0.28, Kruskal–Wallis) and the normal soil (*P* = 0.18). Moreover, significant GC% difference was also observed between the saline soil (55.48% ± 0.32%) and the normal soil (56.69% ± 0.51%) (*P* < 0.01, Wilcoxon signed) but not among six different methods in both the normal soil (*P* = 0.795, Kruskal–Wallis) and the saline soil (*P* = 0.87).

### Alpha Diversity

As rarefaction curves showed, most samples can reach an asymptote, indicating enough sequencing depth (Supplementary Figure S2). Curves of the normal soil were clearly above those of the saline soil, representing different microbial diversities in different soil types.

**FIGURE 1:**
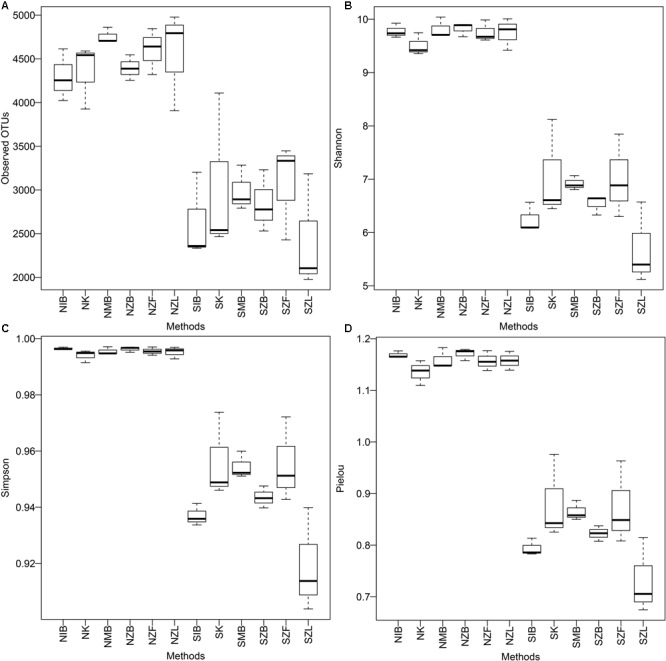
Boxplots of observed OTU number **(A)**, Shannon diversity index **(B)**, Simpson diversity index **(C)**, and Pielou evenness index **(D)** among six extraction methods in both the normal soil and the saline soil SS1. The first two capitals of group names, N and S, represent the normal soil and the saline soil, respectively; other capitals represent the used methods (**Table 2**), K represents the PowerSoil kit.

All alpha diversity indices were significantly different (*P* < 0.001, Wilcoxon signed) between the normal soil and the saline soil (Supplementary Table S4). Boxes of the normal soil were clearly above those of the saline soil (**Figure 1**). These also showed different microbial diversities in different soil types. However, except Pielou evenness index (*P* = 0.031, Kruskal–Wallis) and Simpson index (*P* = 0.036) of the saline soil, we observed no significant difference of other alpha diversity indices (*P* > 0.05) among six different methods in both the two soils. Moreover, boxes of SZL were clearly lower than those of other methods (**Figure 1**); significantly lower Shannon index (*P* = 0.05, Mann–Whitney), Pielou index (*P* = 0.03), and Simpson index (*P* = 0.02) were observed within Zhou’s method with liquid nitrogen grinding step (SZL) than other methods in the saline soil (Supplementary Table S4).

**FIGURE 2:**
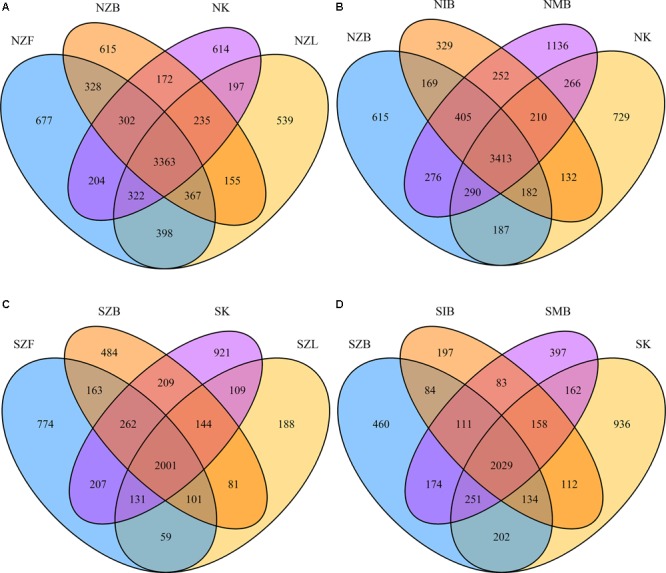
Venn diagrams of OTUs shared by different DNA extraction methods. The OTUs were shared by Zhou’s method with three different physical lysis steps and PowerSoil kit in the normal soil **(A)**, the saline soil **(C)**. The OTUs were shared by three different methods with the same bead beating step and the PowerSoil kit in the normal soil **(B)**, the saline soil **(D)**.

Venn diagrams showed that biases existed among different methods in the same soil at the OTU level (**Figure 2**). Shared OTU percentages were summarized in the Supplementary Table S5. Every method had its unique OTUs with a mean of 11.59% ± 3.33% (s.d.) in the normal soil and 15.01% ± 6.83% in the saline soil (Supplementary Table S5). Also, percentages of unique OTUs were clearly low within SZL, NIB, and SIB groups compared with other groups in both the two soils. The sum of OTU percentages shared by three or four methods were 76.25% ± 3.94% in the normal soil and 73.25% ± 7.99% in the saline soil. Moreover, the IB method achieved clearly lower OTU sum (NIB: 5092) than other methods in the normal soil; except the IB method (SIB: 2968), the ZL method also achieved clearly lower OTU sum (SZL: 2814) than other methods in the saline soil (Supplementary Table S5).

### Prokaryotic Community Compositions

**FIGURE 3:**
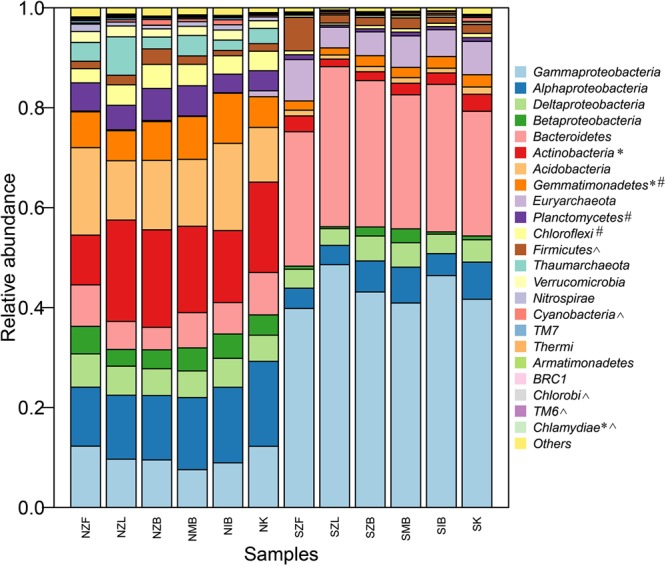
The prokaryotic community composition at the phylum/class level (top 20 most abundant phyla including four proteobacterial classes) of all methods in the saline soil and the normal soil. The phylum labeled with ^∗^ was significantly different (*P* < 0.05, Kruskal–Wallis) among six methods in the normal soil, so was the phylum labeled with # in the saline soil. The phylum labeled with ˆ was unsignificantly different (*P* > 0.05, Wilcoxon signed) between the saline soil and the normal soil.

The prokaryotic community compositions in soils of our present study were similar to previous reports (Hollister et al., 2010; Keshri et al., 2013). Most phyla/classes were significantly different between the two different soils but not among six different methods in the same soil, and the prokaryotic community composition in the normal soil clearly differed from that in the saline soil at the phylum/class level (**Figure 3**). Prokaryotic community compositions of all replicates were also showed (Supplementary Figure S3). Moreover, perMANOVA at OTU level further confirmed significant community difference between two different soils no matter which method was employed but not among six different methods in the same soil (**Table 3**).

**Table 3:** Results of one-way perMANOVA at OTU level between the two soils and among different methods in the same soil.

Group 1	Group 2	Prokaryotic community		KEGG orthology composition	
		*F* value	*P*-value	*F* value	*P*-value
Different soils	all	81.39	**0.0001**	75.35	**0.0001**
	ZL	43.89	**0.0007**	27.43	**0.0003**
	ZF	52.5	**0.0001**	35.12	**0.0001**
	ZB	51.94	**0.0001**	58.67	**0.0001**
	MB	45.82	**0.0001**	51.24	**0.0001**
	IB	17.78	**0.0004**	23.64	**0.001**
	Kit	28.54	**0.0002**	37.84	**0.0001**
Different methods	Normal soil	1.045	0.4117	1.737	0.079
	Saline soil	1.097	0.2994	1.012	0.45

*The significant value was labeled as boldface.*

To further verify variations of each phylum/class in different methods, we calculated and drew boxplots of top six most abundant phyla including four proteobacterial classes (Supplementary Figure S4). All nine phyla/classes differed clearly between the two different soils. For the nine phyla/classes, though only *Actinobacteria* and *Gemmatimonadetes* in the normal soil and *Gemmatimonadetes* in the saline soil were significantly different among different methods within the same soil (*P* < 0.05, Kruskal–Wallis), the relative abundance of each phylum/class can vary greatly among different methods even in the same method (Supplementary Figure S4). The relative abundance of *Bacteroidetes, Euryarchaeota* in the normal soil and *Betaproteobacteria, Actinobacteria, Acidobacteria, Euryarchaeota* in the saline soil varied by a factor of more than five. The six methods also showed different biases: except *Gammaproteobacteria* and *Bacteroidetes*, SZL group had clearly low relative abundance of other seven phyla/classes in the saline soil, no similar trend was observed in NZL group in the normal soil. For the Gram-positive bacteria-*Actinobacteria*, NZF group showed the minimal relative abundance and NZL group showed the maximum in the normal soil (Supplementary Figure S4F).

**FIGURE 4:**
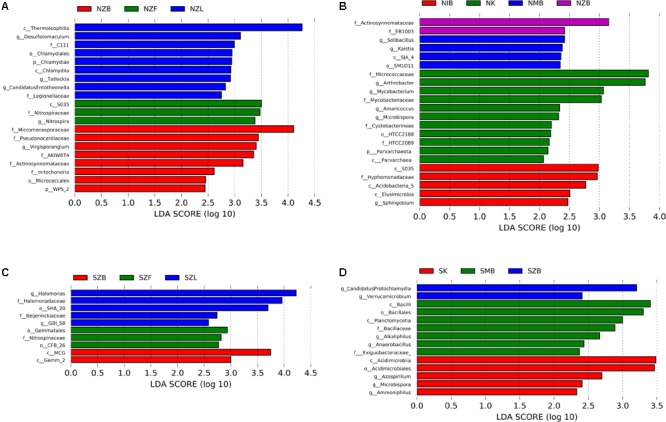
LEfSe analysis with LDA values higher than two among methods with different physical lysis steps in the normal soil **(A)**, the saline soil **(C)** and among different methods with the same bead beating step in the normal soil **(B)**, the saline soil **(D)**. The labels (p, c, o, f, g) before each taxon represented phylum, class, order, family, and genus, respectively.

Top 24 most abundant genera occupied much higher percentage in the saline soil than the normal soil (Supplementary Figure S5), suggesting lower evenness in saline soils (Supplementary Table S4). Top four genera (*KSA1, Marinobacter, Halomonas, Idiomarina*) were all halotolerant or halophilic bacteria in marine environments, and the sum of four genera in SZL group was higher than other groups (Supplementary Figure S5B). The LEfSe analysis has also indicated high relative abundance of *Halomonas* in the SZL group (**Figure 4C**).

Except ISO 11063 method with bead beating in the saline soil (SIB), each method has several indicator taxa with LDA value higher than two even four according to the LEfSe analysis (**Figure 4**). Zhou’s method with bead beating (ZB) was detected with different indicator taxa compared with different methods in the same soil sample (**Figure 4**). The family *Actinosynnemataceae* was detected within NZB group in both **Figures 4A,B**, suggesting *Actinosynnemataceae* may be an indicator taxon of the ZB method in the normal soil.

### Gram-Positive Bacteria Proportions

We observed significant differences of Gram-positive bacteria between the saline soil (0.032 ± 0.012) and the normal soil (0.184 ± 0.051) (*P* < 0.01, Wilcoxon signed) (Supplementary Figure S7). No significant difference was observed among six different methods in the same soil (*P* = 0.08 in the normal soil, *P* = 0.51 in the saline soil, Kruskal–Wallis). The box of NZF was clearly lower than other boxes in the normal soil (Supplementary Figure S7), suggesting the freezing and thawing method may be less effective for Gram-positive bacteria than other two physical methods. The box of NIB was also relatively low in the normal soil. A clear outlier was observed within SZF group (Supplementary Figure S7B). Moreover, most Gram-positive bacteria were assigned to *Actinobacteria* in both two soils (Supplementary Figure S8B); most Gram-negative bacteria in the saline soil was assigned to *Proteobacteria* and *Bacteroidetes* (Supplementary Figure S8A), being consistent with the prokaryotic community of the saline soil (**Figure 3**). The box of SZL was lower than other boxes in the saline soil (Supplementary Figure S7B) and we observed no *Actinobacteria* within SZL group (Supplementary Figure S7B).

### Beta Diversity

**FIGURE 5:**
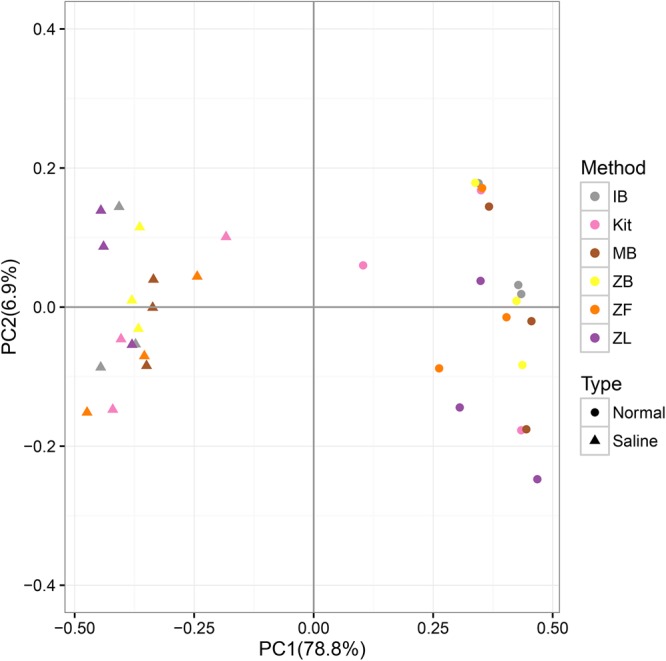
PCoA (principal coordinate analyses) based on the weighted Unifrac distance calculated with the normalized OTU table and a corresponding phylogenetic tree.

The first axis explained 78.8% of community variations, and samples can be well separated along the first axis based on their soil types; the second axis explained only 6.9% of community variations according to different extraction methods (**Figure 5**). UPGMA clustering based on the genus-level community also showed that samples can be well clustered according to their soil types but not different methods (Supplementary Figure S6). These suggested good reproducibility of all DNA extraction methods in the whole community.

### Phylogenetic Analysis

Significantly lower MNTD (*P* = 0.002, Wilcoxon signed) and significantly higher NTI values (*P* < 0.001) were observed in the normal soil than the saline soil (Supplementary Figure S9). These indicated different phylogenetic clustering extents in different soil types and the stronger phylogenetic clustering extent in the normal soil than the saline soil. Also, we observed no significant difference of both MNTD (*P* = 0.63 in the normal soil, *P* = 0.64 in the saline soil; Kruskal–Wallis) and NTI (*P* = 0.49 in the normal soil; *P* = 0.29 in the saline soil) among six different methods in the same soil. However, the phylogenetic clustering extent can also change greatly among different extraction methods even within the same method (Supplementary Figure S9), though the difference was unsignificant.

### Functional Prediction With PICRUSt

**FIGURE 6:**
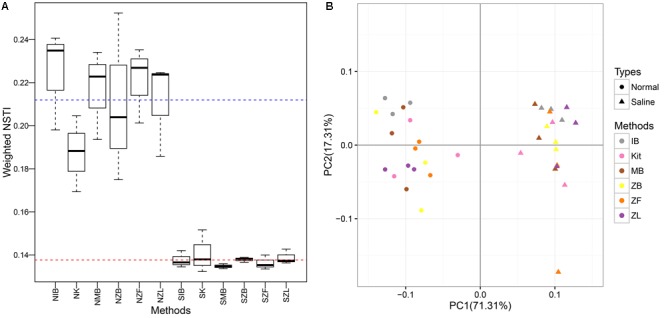
Boxplots of weighted NSTI (Nearest Sequenced Taxon Index) values **(A)** and PCoA based on the Bray-Curtis distance calculated with the predictive KEGG Orthology table **(B)**. The blue and red dotted line represented the mean of NSTI values in the normal soil and in the saline soil, respectively.

The NSTI values were clearly higher in the normal soil (0.212 ± 0.024) than the saline soil (0.138 ± 0.004), indicating higher predictive accuracy in the saline soil (**Figure 6**). Significant differences were observed between the normal soil and the saline soil (*P* < 0.001, Wilcoxon signed) but not among six different methods in the same soil (*P* = 0.51 for the normal soil, *P* = 0.54 for the saline soil; Kruskal–Wallis). The predictive KEGG pathways at level two were summarized in Supplementary Figure S10, and clear differences can be observed between the normal soil and the saline soil. Samples can be well separated based on their soil types along the first axis (71.3%), but they cannot be well clustered together according to different methods along the second axis (17.3%). Further perMANOVA confirmed the significant functional composition difference between the saline soil and the normal soil no matter which method was used, and no significant difference was observed among six different methods in the same soil (**Table 3**).

## Discussion

In the present study, we employed several direct DNA extraction methods to optimize extraction methods for saline soils and estimated soil microbiome biases associated with different DNA extraction methods. The saline soil SS1 for further sequencing were saline and alkaline; the saline soil has much higher salinity, lower TOC and lower WC than the normal soil, associated with previous reports (Keshri et al., 2013; Xie et al., 2017).

Because of high salinity and low biomass in saline soils, DNA was difficult to extract from saline soils or sediments (Keshri et al., 2013; Natarajan et al., 2016; Xie et al., 2017). The soil washing pretreatment can substantially improve DNA quantity and quality from contaminated or high organic sediments (Fortin et al., 2004; Fang et al., 2014). Though saline soils were uncontaminated and organic contents in them were not high, huge amounts of salts in saline especially hypersaline soils may greatly change the original state of DNA extraction buffers. Therefore, we applied the soil washing pretreatment with PBS to prevent salt interferences. The quality and quantity of DNA increased clearly after PBS washing (Supplementary Table S2). For most hypersaline soil samples in our previous study, detectable DNA amount even cannot be achieved without PBS washing pretreatment (Xie et al., 2017). In this study, the soil washing pretreatment was applied within all DNA extraction methods except ZL method in the saline soil.

Previous reports have observed that more DNA was achieved with manual methods than the commercial kit (Cruaud et al., 2014; Natarajan et al., 2016). However, crude DNA achieved with manual methods cannot be directly PCR-amplified for further sequencing (Zhou et al., 1996; Arbeli and Fuentes, 2007), and crude DNA yields were often overestimated based on UV measurements (Kuhn et al., 2017). Therefore, we employed additional purification procedures. We observed the purified DNA quantity achieved with manual methods was significantly lower than PowerSoil kit in both the two soils, suggesting the low recovery rate of additional purification procedures (Arbeli and Fuentes, 2007; Lever et al., 2015). Moreover, purified DNA solutions achieved with five manual methods has higher OD_260_/OD_230_ ratios and lower OD_260_/OD_280_ ratios than PowerSoil kit, agreed with a previous report (Cruaud et al., 2014). That suggested manual methods did better in clearing away the humus, and the commercial kit was more efficient at reducing protein contaminations (Schultz et al., 1994; Griffiths et al., 1997).

The DNA quantity was significantly different between the two soils, corroborating different DNA quantity achieved from different soil types (Cruaud et al., 2014; Wust et al., 2016). The DNA quantity was also different among different DNA extraction methods, being consistent with previous reports (Wust et al., 2016; Gupta et al., 2017), though the difference was unsignificant among five manual methods in the present study.

Accorded with previous reports (Cruaud et al., 2014; Wust et al., 2016), numbers of raw tags and effective tags were unsignificantly different between the two soils or among six different DNA extraction methods, suggesting that the amplification efficiency was independent of soil type, DNA quality and quantity (Cruaud et al., 2014). However, we observed significant effective ratio (effective tags/raw tags) and GC% differences between the two soils but not among different methods; that also agreed with previous reports (Haas et al., 2011; Cruaud et al., 2014). The results indicated that DNA extraction methods have no effect on the formation of low-quality or chimera reads, and the chimera formation or GC% depended on soil types and their associated microbial community.

All alpha diversity indices were significantly different between the two soils, and the microbial diversity was much lower in the saline soil than the normal soil (**Figure 1**, Supplementary Figure S2, and Supplementary Table S4), being consistent with previous reports (Keshri et al., 2013; Xie et al., 2017). Also, except Pielou evenness index and Simpson diversity index in the saline soil, other alpha diversity indices were all unsignificantly different among six DNA extraction methods in the same soil. That indicated evenness indices may be more easily affected by DNA extraction methods than richness indices in the saline soil. That was probably due to low evenness in the saline soil compared with the normal soil (Supplementary Table S4 and Supplementary Figure S5), the variation of a single species caused by DNA extraction methods had more effects on the whole community. Previous reports have also observed no significant difference of microbial diversities between two extraction methods and the microbial diversities were unrelated with achieved DNA quantities (Cruaud et al., 2014).

The ISO 11063 method has less unique OTUs and lower OTU sum than other methods according to Venn diagrams (**Figure 2** and Supplementary Table S5); the Gram-positive bacteria proportion in NIB group was also relatively low in the normal soil (Supplementary Figure S7B). Previous studies have reported that the ISO 11063 method underestimated the rRNA gene abundance in soil microbiomes (Terrat et al., 2015; Wust et al., 2016). Compared with other two methods (ZB, MB), the ISO 11063 method lacked the enzyme lysis step (**Table 2**). We inferred this factor mainly caused the low effectiveness of ISO 11063 method. Therefore, the physical lysis step should be combined with the enzyme lysis step.

We observed no significant alpha diversity difference caused only by physical lysis methods in the normal soil (**Figure 1**), though less Gram-positive bacteria were achieved with the freezing and thawing method. Also, the high proportion of Gram-positive bacteria within NZL group (Supplementary Figures S4F, S7B) suggested the liquid nitrogen grinding step was effective for Gram-positive bacteria in the normal soil, but lower alpha diversity indices (**Figure 1** and Supplementary Table S4), less unique OTUs (**Figure 2** and Supplementary Table S5), and lower proportion of Gram-positive bacteria (Supplementary Figure S7B) were observed within SZL group than other methods in the saline soil. Because it is the only method with no soil washing pretreatment, we referred that the soil washing pretreatment can increase not only DNA quality and quantity but also the achieved microbial diversity in saline soils. Though alpha diversity indices were significantly low within SZL group, the total relative abundance of halotolerant or halophilic bacteria was high within the group, suggesting some halotolerant or halophilic bacteria may lyse during the soil washing pretreatment. By summarizing our present and previous studies (Xie et al., 2017), we recommend soil washing pretreatment should be applied for saline soils, and the pretreatment was especially crucial for hypersaline soils that cannot be achieved with detectable DNA amounts without the pretreatment. High proportion of halophilic microorganisms can still be achieved after PBS washing within hypersaline soils in our previous study (Xie et al., 2017).

Gram-positive bacteria were harder to lyse than Gram-negative bacteria (Frostegard et al., 1999). The DNA extraction efficiency was based on lysis extents of Gram-positive bacteria in soils (Wust et al., 2016; Costea et al., 2017). Therefore, we predicted proportions of Gram-positive and Gram-negative bacteria in each sample (Supplementary Figures S7, S8). We observed no significant correlation (*P* > 0.1) between Gram-positive bacteria proportions and alpha diversity indices in the normal soil, but significant positive correlations were observed between Gram-positive bacteria proportions and observed OTU number (*r* = 0.76, *P* < 0.01), Shannon index (*r* = 0.63, *P* = 0.006), Pielou index (*r* = 0.54, *P* = 0.02) without the outlier SZF-A in the saline soil. Previous reports have also observed positive correlations between Gram-positive bacteria proportions and microbial diversities (Wust et al., 2016; Costea et al., 2017).

Only microbial diversity indices were insufficient to evaluate the biases associated with different DNA extraction methods (Terrat et al., 2015; Zielinska et al., 2017). Therefore, we also estimated biases of the microbial community and predictive KEGG functional composition in the present study. We observed significant community differences between the two soils but not among different extraction methods in the same soil (**Table 3, Figure 3**, and Supplementary Figure S6). Samples can be well separated based on their soil types but not the employed extraction methods according to PCoA results (**Figure 5**). These indicated that though deviations existed in different DNA extraction methods, the soil type still determined mainly the whole community composition(Terrat et al., 2015; Santos et al., 2017; Sinha et al., 2017). The functional composition was similar to the above taxonomic community (**Table 3, Figure 6**, and Supplementary Figure S10), suggesting that the soil type also determined primarily functional compositions. That agreed with a recent report the between-subject variability was greater than DNA extraction effects for human fecal samples (Costea et al., 2017).

Though the whole taxonomic community and functional compositions can well be separated according to their soil types, it can deviate results a lot when concentrating on the exact relative abundance of a certain taxon or a certain diversity value (**Figure 1** and Supplementary Figures S4, S7): several phyla/classes can vary by a factor of more than five. Previous studies have also observed the deviations (Cruaud et al., 2014; Birtel et al., 2015; Zielinska et al., 2017). The relative abundance of Gram-positive bacteria, *Actinobacteria*, varied by a factor of up to ten in a previous report (Wust et al., 2016), and *Actinobacteria* was significantly different among six extraction methods in the normal soil of the present study. Except *Actinobacteria, Gemmatimonadetes* were also significantly different among six methods in both the normal soil and the saline soil. Moreover, though only three taxa had LDA values more than four, the LEfSe analysis detected different biomarkers within different methods, indicating the extraction method can bias the LEfSe analysis to some extent.

## Conclusion

The freezing and thawing step was less effective for Gram-positive bacteria than bead beating and liquid nitrogen grinding in the normal soil. For hypersaline soils, the soil washing pretreatment should be applied to improve the success ratio of DNA extraction, and the liquid nitrogen grinding was inapplicable after soil washing. Therefore, we recommend bead beating or liquid nitrogen grinding for normal soils and bead beating for hypersaline soils. Also, physical lysis steps should be combined with enzyme lysis steps to improve the DNA extraction efficiency. Unique species existed in every method (**Figure 2** and Supplementary Table S5). A certain method may be inadequate in soil microbiome studies, and we recommend to pool DNA solutions achieved from different methods even replicates of the same method together for further sequencing. These results can provide references for the DNA extraction from saline soils and experiment designs in soil microbiome studies.

## Author Contributions

KX and HH designed the research. KX collected saline soil samples. XZ measured soil physical and chemical properties. KX and XW extracted DNA from soils. KX and YD analyzed the sequencing data. KX wrote the paper. GK and LB helped reviewing and modifying the paper.

## Conflict of Interest Statement

The authors declare that the research was conducted in the absence of any commercial or financial relationships that could be construed as a potential conflict of interest.
