# Supplementary material for: Biases in Prokaryotic Community Amplicon Sequencing Affected by DNA Extraction Methods in Both Saline and Non-saline Soil
Source: Front Microbiol. 2018 Aug 3;9:1796. doi: 10.3389/fmicb.2018.01796 (PMC6088177; doi:10.3389/fmicb.2018.01796)
Supplement: Supplementary file 1 [file Data_Sheet_1.PDF]

Supplementary Material

Biases in Prokaryotic Community Amplicon Sequencing Affected by DNA Extraction Methods in both Saline and Non-saline Soil

Kehui Xie[1,2]†, Yong Deng[1,2]†, Xuze Zhang[3], Xueting Wang[1,2], Guangbo Kang[1,2], Liang Bai[1,2] and He Huang[1,2]*

[1]Department of Biochemical Engineering, School of Chemical Engineering and Technology, Tianjin University, Tianjin, China; *[2]Key Laboratory of Systems Bioengineering, Ministry of Education, Tianjin University, Tianjin, China*; *[3]School of Chemistry and Chemical Engineering, Qinghai University for Nationalities, Xining, People's Republic of China.*

***Correspondence:**

He Huang

huang@tju.edu.cn.

†K.X. and Y. D. contributed equally to this work.

Supplementary Tables

Table S1. The quantity and quality of achieved DNA

Replicates	OD_{260}/OD_{280}	OD_{260}/OD_{230}	Yield (ug DNA/g soil)
NZF-A	1.634	2.071	2.90
NZF-B	1.522	2.694	3.68
NZF-C	1.656	3.598	4.15
NZL-A	1.764	1.699	7.59
NZL-B	1.777	2.355	3.95
NZL-C	1.724	1.717	3.06
NZB-A	1.79	2.117	3.67
NZB-B	1.806	2.265	2.93
NZB-C	1.765	2.101	4.55
NMB-A	1.789	2.044	3.58
NMB-B	1.875	1.906	5.63
NMB-C	1.583	2.159	5.12
NIB-A	1.781	2.172	6.43
NIB-B	1.832	1.533	5.27
NIB-C	1.842	1.961	5.66
NK-A	1.937	1.618	23.88
NK-B	1.879	1.4	27.48
NK-C	1.812	1.426	20.73
SZF-A	1.315	2.303	2.34
SZF-B	1.626	2.215	2.82
SZF-C	1.768	4.073	2.39
SZL-A	1.83	3.288	2.85
SZL-B	1.786	2.582	3.10
SZL-C	1.82	3.752	2.48
SZB-A	1.862	3.578	2.89
SZB-B	1.86	2.743	3.27
SZB-C	1.475	1.433	3.28
SMB-A	1.852	2.137	4.59
SMB-B	1.797	3.548	2.26
SMB-C	1.784	2.396	2.43
SIB-A	1.87	2.252	4.04
SIB-B	1.246	1.651	5.72
SIB-C	1.745	1.354	3.64
SK-A	1.847	1.534	9.99
SK-B	1.906	1.629	11.37
SK-C	1.754	1.383	12.61

Abbreviations: N, normal soil; S, saline soil; ZF, ZL, ZB, MB, IB: the methods summarized in Table 2; K represents the PowerSoil kit. Each group has three replicates (A, B, C).

Table S2. The achieved DNA quality and quantity from saline soils with or without soil washing using the PowerSoil kit

Samples	Replicates	without soil washing			with soil washing		
		OD_{260}/ OD_{280}	OD_{260}/ OD_{230}	Yield (ug DNA/g soil)	OD_{260}/ OD_{280}	OD_{260}/ OD_{230}	Yield (ug DNA/g soil)
SS1	1	1.735	1.124	7.67	1.847	1.534	9.99
	2	2.002	1.185	7.76	1.906	1.629	11.37
	3	1.923	1.006	7.13	1.754	1.383	12.61
SS2	1	1.777	0.94	3.08	1.85	1.279	6.81
	2	1.849	0.835	3.08	1.895	1.31	6.55
	3	1.93	0.714	4.21	1.941	1.143	6.53

Table S3. Statistics of sequencing data

SampleID	Accession no.	Raw Tags	Effective tags	Effective ratio	Average length	GC%	Q20
NIB-A	SRS2717108	93226	87258	0.94	253	57.11	99.05
NIB-B	SRS2717107	97287	90440	0.93	253	57.05	99.06
NIB-C	SRS2717110	95627	87251	0.91	253	56.27	99.04
NMB-A	SRS2717109	80381	74264	0.92	253	56.89	98.92
NMB-B	SRS2717112	89775	74262	0.83	253	57.51	98.96
NMB-C	SRS2717111	95088	85718	0.90	253	55.91	99.08
NZB-A	SRS2717113	77387	68911	0.89	253	56.78	99.12
NZB-B	SRS2717114	94813	86859	0.92	253	57.36	98.93
NZB-C	SRS2717116	80178	72933	0.91	253	56.46	99.08
NZF-A	SRS2717115	93780	76586	0.82	254	56.69	98.97
NZF-B	SRS2717100	78007	71332	0.91	253	56.52	98.48
NZF-C	SRS2717099	83577	75428	0.90	253	55.67	99
NZL-A	SRS2717102	81216	75921	0.93	253	56.77	99.04
NZL-B	SRS2717101	81280	74479	0.92	253	57.05	98.51
NZL-C	SRS2717104	80219	73045	0.91	253	56.23	99.02
NK-A	SRS2717103	86116	72044	0.84	253	57.3	98.99
NK-B	SRS2717106	89462	81146	0.91	253	56.63	99.03
NK-C	SRS2717105	84543	77259	0.91	253	56.21	99.05
SIB-A	SRS2717092	89547	83358	0.93	253	55.22	98.61
SIB-B	SRS2717091	94706	88493	0.93	253	55.89	98.6
SIB-C	SRS2717082	95803	89765	0.94	253	55.56	99.12
SMB-A	SRS2717081	89222	81785	0.92	253	55.78	98.63
SMB-B	SRS2717083	79437	73081	0.92	253	55.51	98.55
SMB-C	SRS2717084	98065	91359	0.93	253	55.38	99.04
SZB-A	SRS2717085	82524	75740	0.92	253	55.24	98.72
SZB-B	SRS2717086	85755	79091	0.92	253	55.38	98.51
SZB-C	SRS2717087	90029	84299	0.94	253	55.62	99.07
SZF-A	SRS2717088	76944	64820	0.84	253	54.87	98.83
SZF-B	SRS2717089	94516	88243	0.93	253	55.86	98.72
SZF-C	SRS2717090	80985	75645	0.93	253	55.48	99.05
SZL-A	SRS2717098	88412	83025	0.94	253	55.18	98.59
SZL-B	SRS2717097	94299	88100	0.93	253	55.25	98.72
SZL-C	SRS2717096	79538	74653	0.94	253	55.55	99.1
SK-A	SRS2717095	80621	74967	0.93	253	56.11	98.57
SK-B	SRS2717094	93555	87226	0.93	253	55.72	98.62
SK-C	SRS2717093	91117	84095	0.92	253	55.02	99.11

Table S4. Alpha diversity indices of sequencing data

SampleID	Observed OTUs	Shannon	Pielou	Chao1	Simpson	Goods_coverage
NIB-A	4023.1	9.66	1.16	5495.3	1.00	0.98
NIB-B	4254.7	9.74	1.17	5977.8	1.00	0.98
NIB-C	4614.3	9.93	1.18	6538.9	1.00	0.97
NMB-A	4703.5	9.71	1.15	7190.7	0.99	0.97
NMB-B	4706.2	9.71	1.15	7187.7	0.99	0.97
NMB-C	4860.9	10.04	1.18	6847.5	1.00	0.97
NZB-A	4388.2	9.89	1.18	6018.9	1.00	0.98
NZB-B	4254.5	9.67	1.16	5748.5	1.00	0.98
NZB-C	4546.5	9.90	1.18	6373.2	1.00	0.98
NZF-A	4320.9	9.67	1.16	5812.8	1.00	0.98
NZF-B	4641.3	9.61	1.14	6504.4	0.99	0.97
NZF-C	4846.3	9.99	1.18	6781.4	1.00	0.97
NZL-A	3906.3	9.42	1.14	5323.1	0.99	0.98
NZL-B	4794	9.81	1.16	6511.2	1.00	0.97
NZL-C	4978.1	10.01	1.18	7098.3	1.00	0.97
NK-A	3925.9	9.42	1.14	5496.4	0.99	0.98
NK-B	4542.9	9.75	1.16	6536.0	1.00	0.97
NK-C	4590.3	9.36	1.11	6519.3	0.99	0.97
SIB-A	2333	6.09	0.79	3889.3	0.94	0.98
SIB-B	2358.7	6.08	0.78	3909.2	0.93	0.98
SIB-C	3202.5	6.57	0.81	5180.5	0.94	0.98
SMB-A	2892.3	7.07	0.89	4439.5	0.96	0.98
SMB-B	2792	6.81	0.86	4451.4	0.95	0.98
SMB-C	3283.7	6.88	0.85	5369.9	0.95	0.98
SZB-A	2530.6	6.33	0.81	4062.6	0.94	0.98
SZB-B	2777.1	6.64	0.84	4430.5	0.95	0.98
SZB-C	3231.4	6.65	0.82	5034.0	0.94	0.98
SZF-A	3447.1	7.85	0.96	5118.6	0.97	0.98
SZF-B	2428.5	6.30	0.81	3905.6	0.94	0.98
SZF-C	3333.6	6.88	0.85	5239.1	0.95	0.98
SZL-A	1977	5.12	0.67	3545.0	0.90	0.99
SZL-B	2104.2	5.40	0.71	3574.4	0.91	0.98
SZL-C	3185.4	6.57	0.81	4962.3	0.94	0.98
SK-A	2540.5	6.61	0.84	4078.4	0.95	0.98
SK-B	2465.3	6.45	0.83	3856.2	0.95	0.98
SK-C	4108.7	8.12	0.98	6078.8	0.97	0.98

Table S5. Percentages of OTUs shared by different DNA extraction methods

Group	Methods	UniPer (%)	TwoPer (%)	ThrPer (%)	FourPer (%)	OTU sum
A	NZF	11.36	15.60	16.62	56.42	5961
	NZB	11.11	11.83	16.33	60.74	5537
	NZL	9.67	13.45	16.57	60.31	5576
	NK	11.35	10.59	15.88	62.17	5409
B	NZB	11.11	11.41	15.84	61.64	5537
	NIB	**6.46**	10.86	15.65	67.03	**5092**
	NMB	18.18	12.71	14.48	54.63	6248
	NK	13.48	10.82	12.61	63.10	5409
C	SZF	20.93	11.60	13.36	54.11	3698
	SZB	14.05	13.15	14.72	58.08	3445
	SZL	**6.68**	8.85	13.36	71.11	**2814**
	SK	23.12	13.18	13.48	50.23	3984
D	SZB	13.35	13.35	14.40	58.90	3445
	SIB	**6.64**	9.40	15.60	68.36	**2968**
	SMB	11.80	12.45	15.45	60.30	3365
	SK	23.49	11.95	13.63	50.93	3984

Abbreviations: the UniPer represented the percentage of unique OTUs in each method; the TwoPer, ThrPer, and FourPer represented the percentage of OTUs shared by two methods, three methods, and four methods, respectively; OTU sum represents the sum of OTUs within each group; the clearly low values was labeled as boldface.

Supplementary Figures

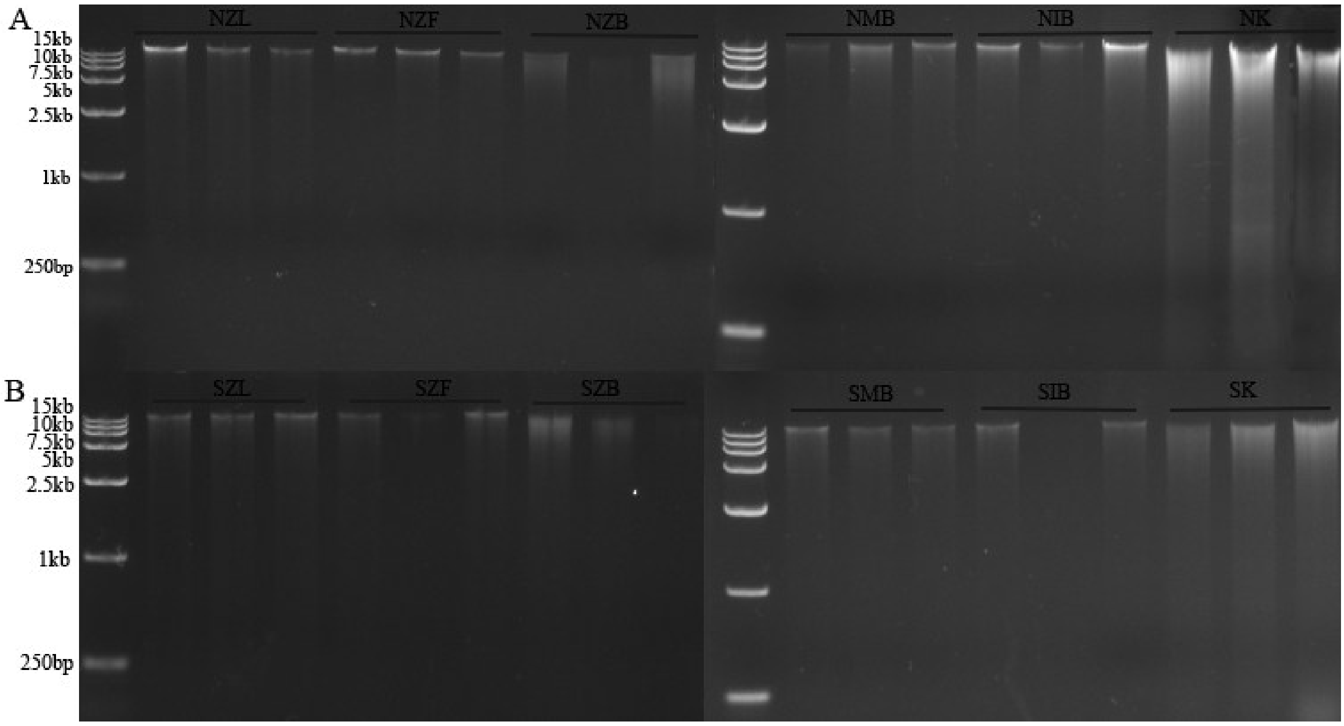

Figure S1. The agarose gel electrophoresis maps of extracted DNA from the normal soil (A) and the saline soil SS1 (B)

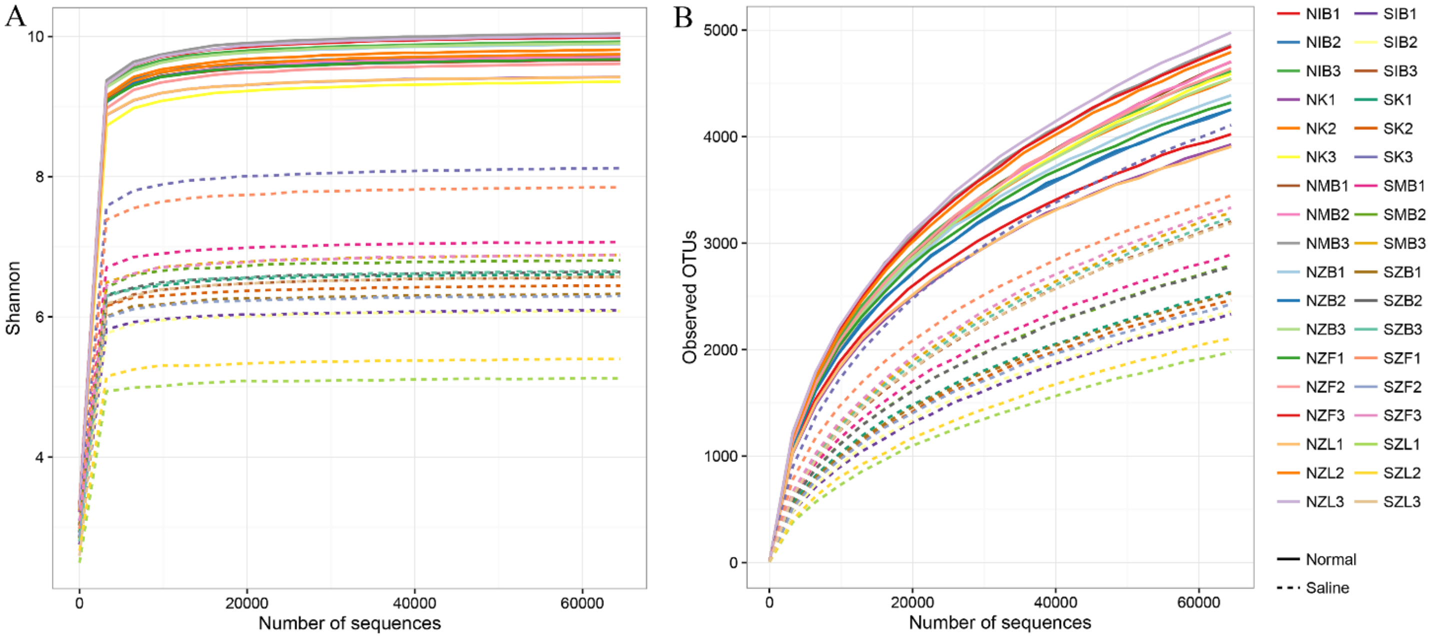

Figure S2. Rarefaction curves for Shannon diversity index (A), observed OTUs (B) of all replicates in two soils. The linetype represented different soil types with the solid line representing the normal soil and the dotted line representing the saline soil.

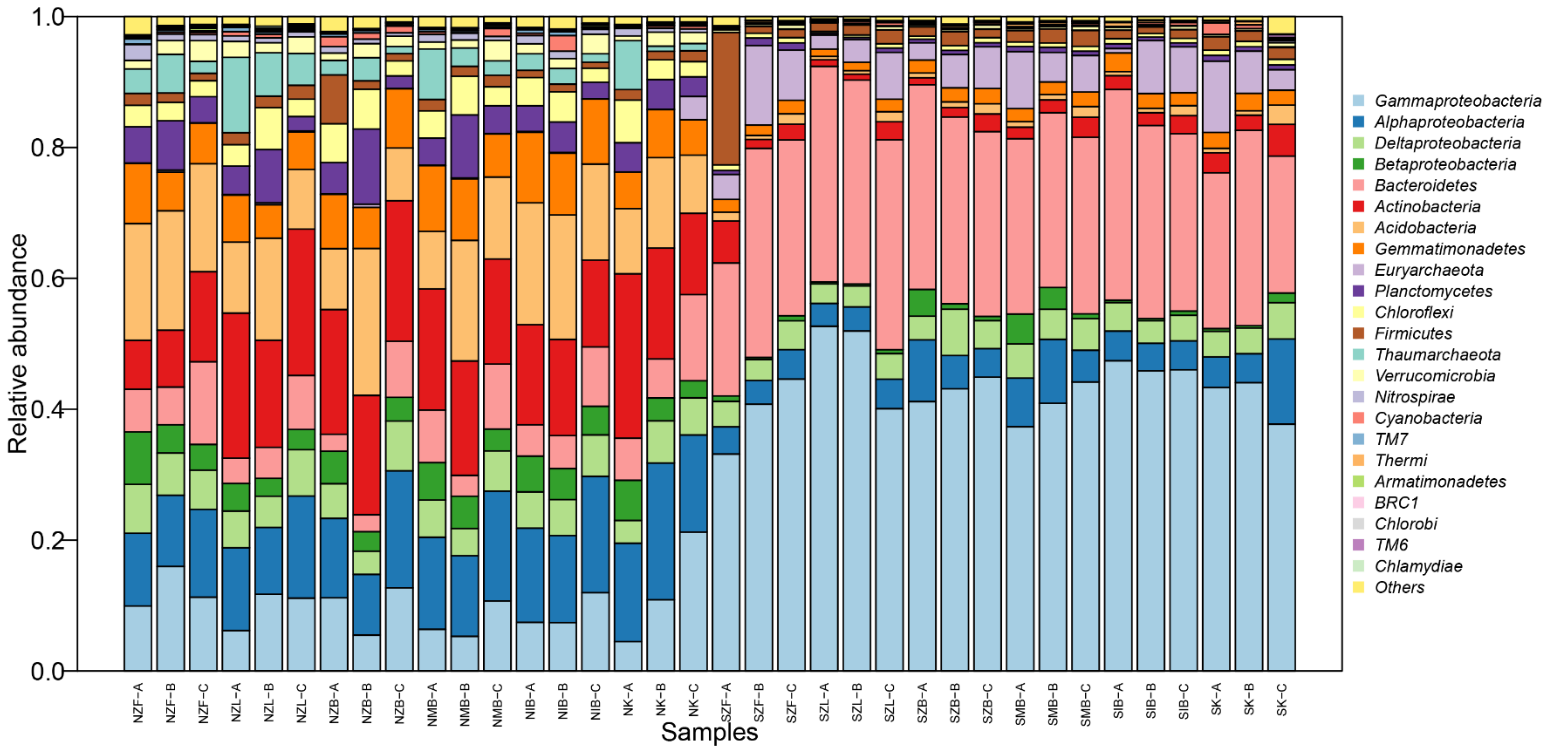

Figure S3. The prokaryotic community composition at the phylum/class level (top 20 most abundant phyla including four proteobacterial classes) of all replicates in the normal soil and the saline soil.

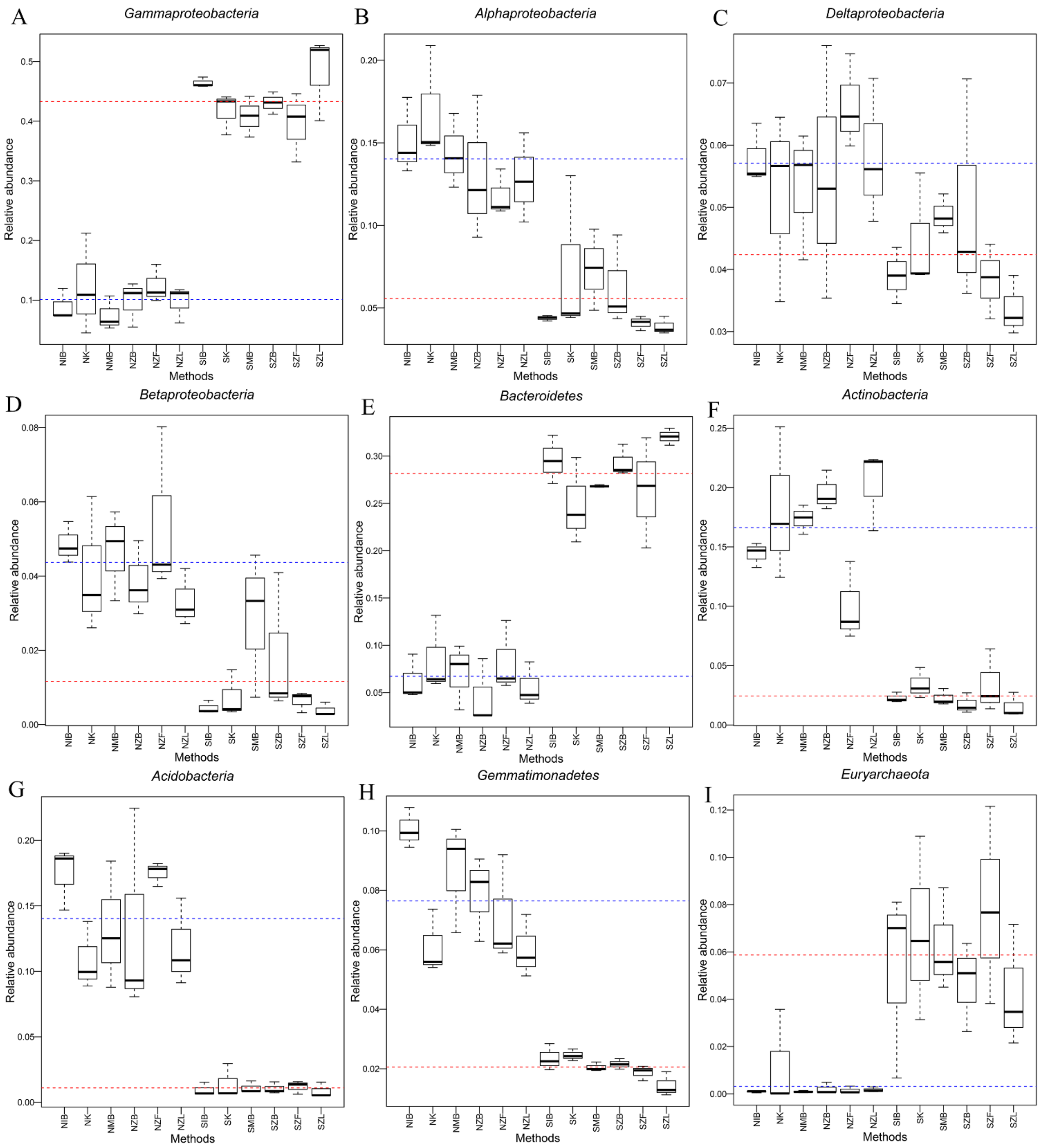

Figure S4. Boxplots of top six most abundant phyla (including four proteobacterial classes) within six different methods in each soil. The blue dotted line was the mean relative abundance value of all replicates in the normal soil, so was the red dotted line in the saline soil.

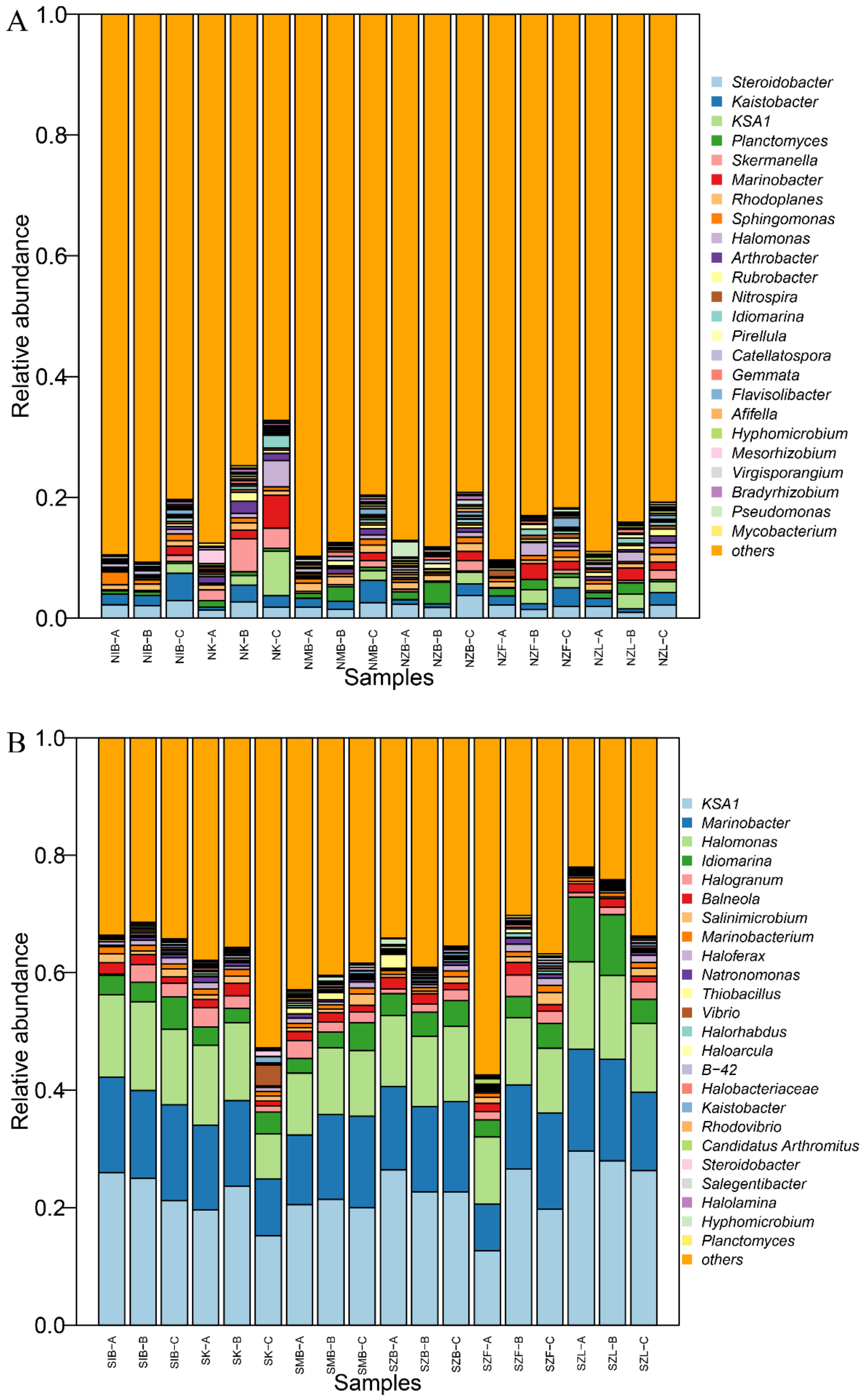

Figure S5. The prokaryotic community composition at the genus level (top 24 most abundant genus) of all replicates in the normal soil (A) and the saline soil (B).

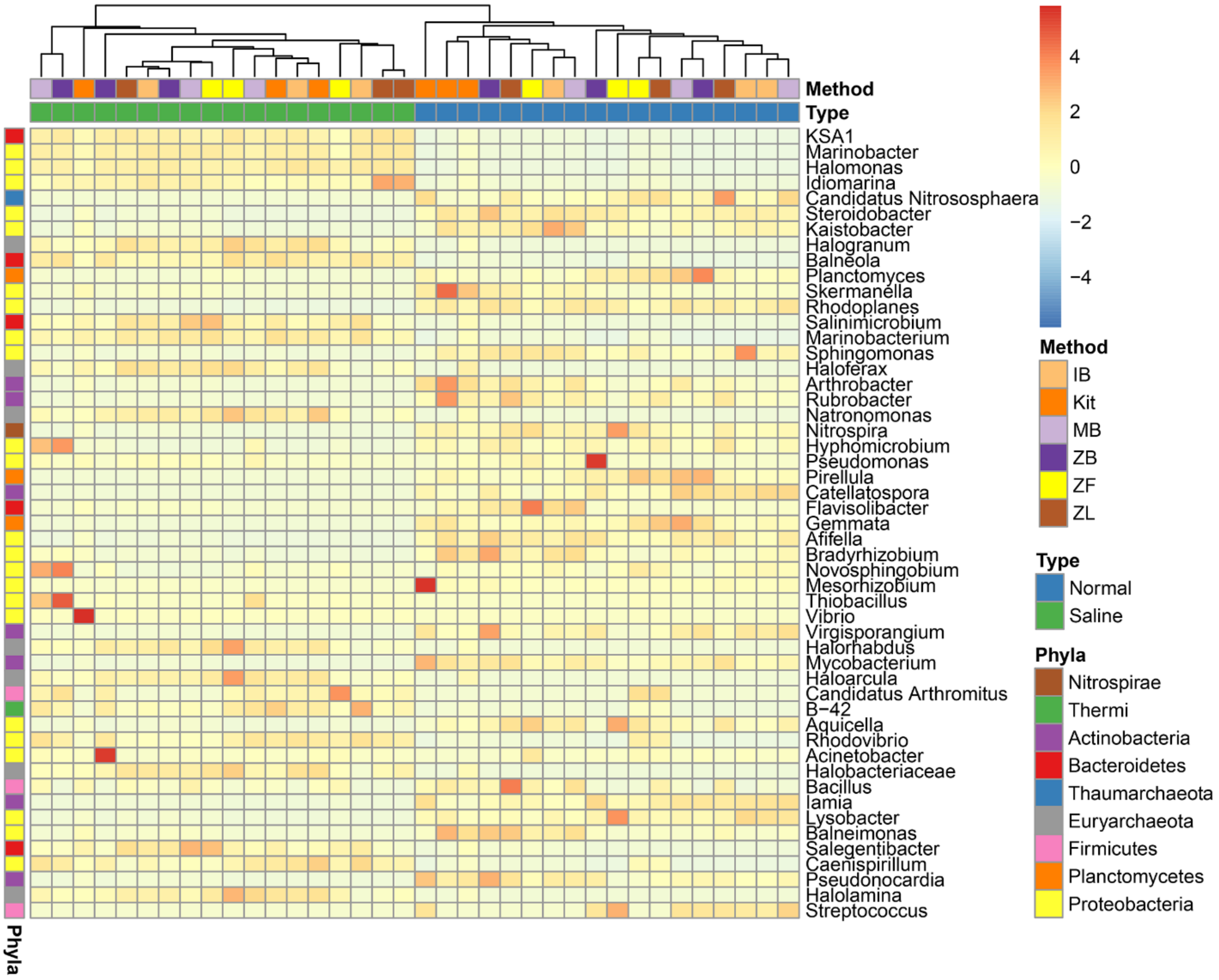

Figure S6. Heat map based on the community composition at the genus level. The relative abundance of each genus was subjected to z-transformation ([x-mean]/[standard deviation]). The clustering analysis of all samples was based on the Pearson correlation distance with unweighted pair-group method with arithmetic means (UPGMA).

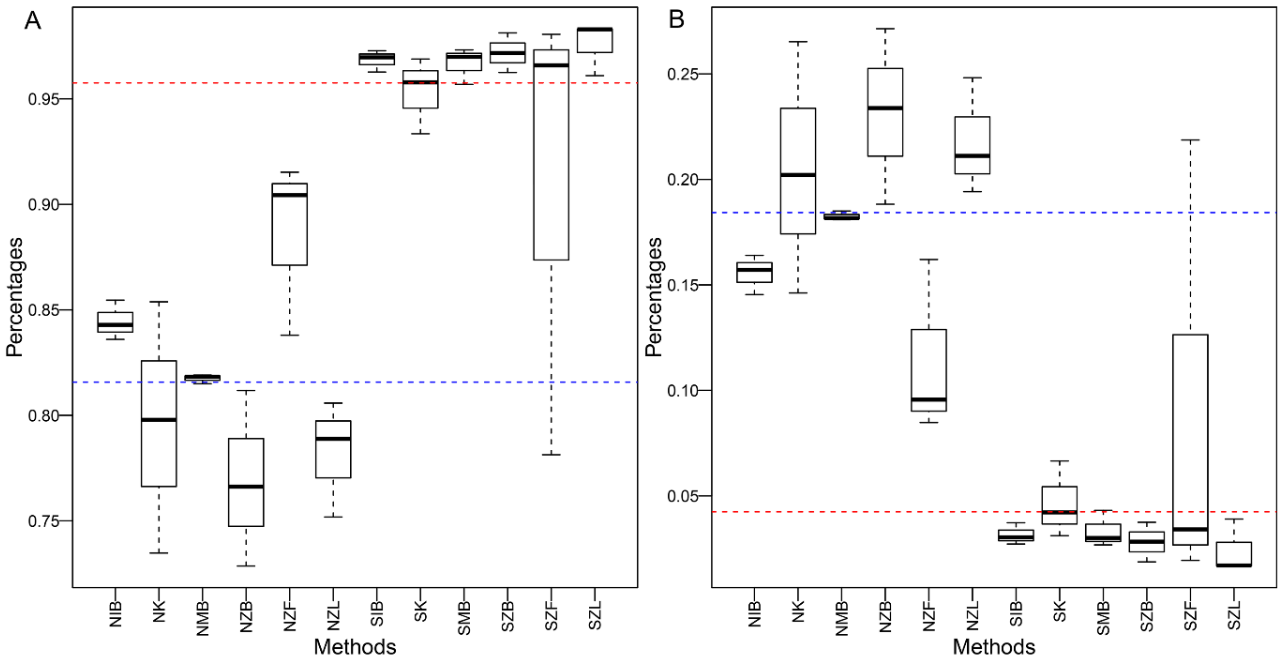

Figure S7. Boxplots of Gram-negative bacteria proportions (A) and Gram-positive bacteria proportions (B) of each method in both the normal soil and the saline soil.

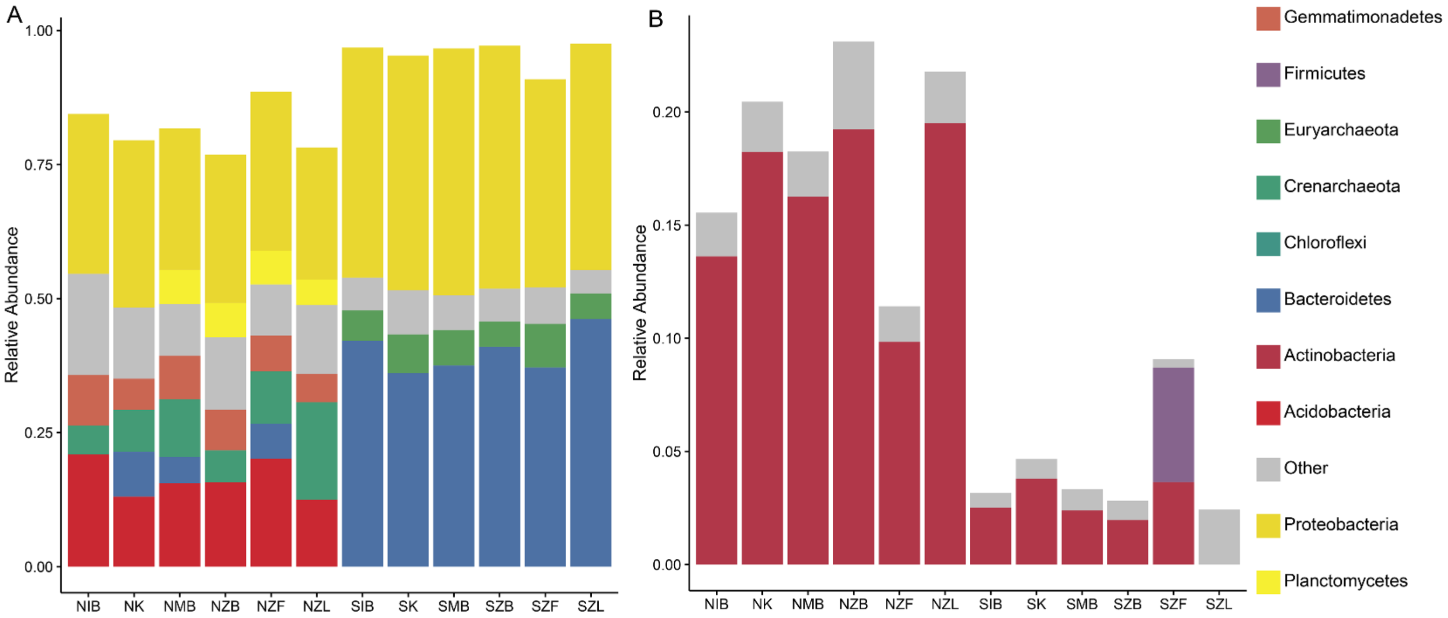

Figure S8. The community compositions of Gram-negative bacteria (A) and Gram-positive bacteria (B) at the phylum level in all methods of both the normal soil and the saline soil.

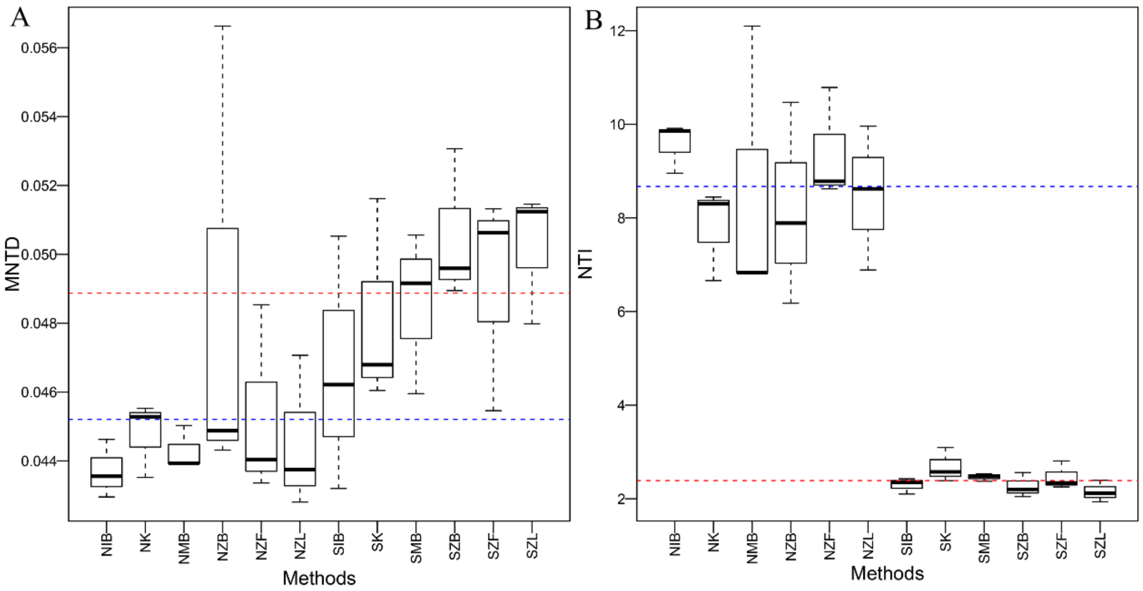

Figure S9. Boxplots of phylogenetic data including MNTD (mean nearest taxon distance) (A) and NTI (net relatedness index) (B). The blue and red dotted line were the means of all replicates in the normal soil and the saline soil, respectively.

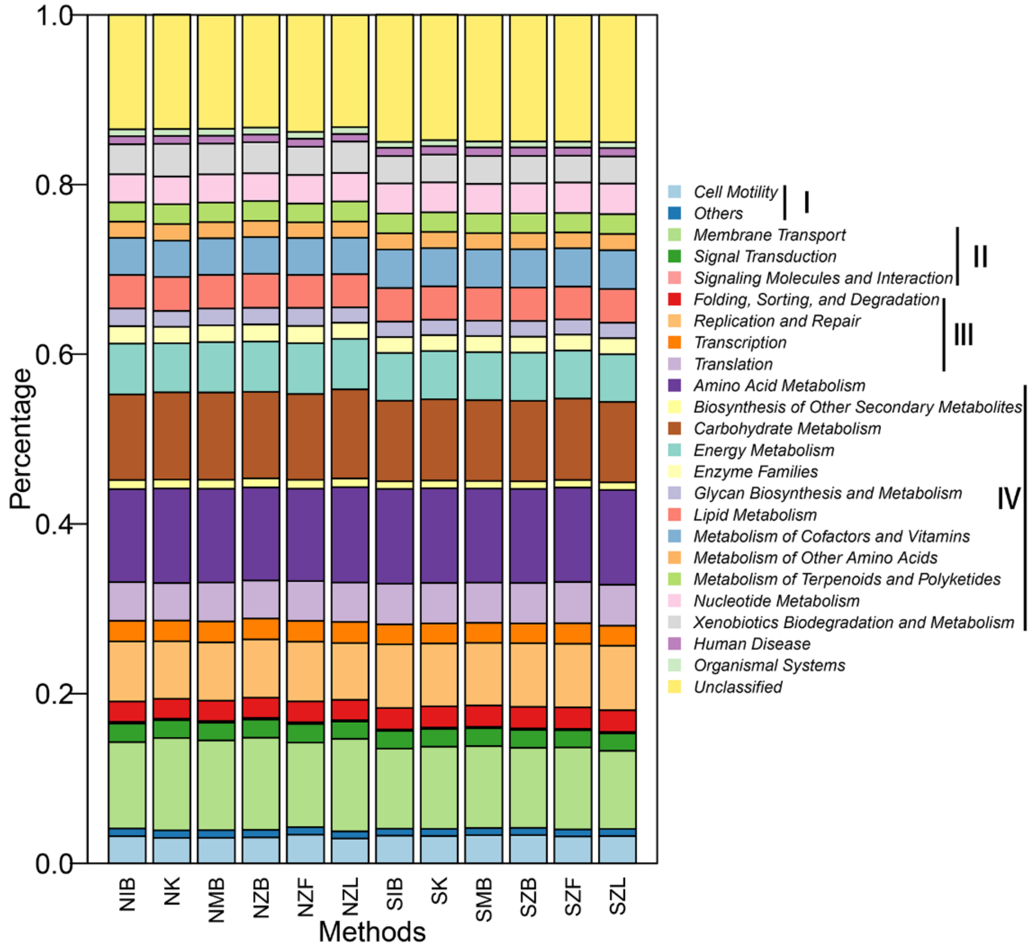

Figure S10. The predictive KEGG pathways at level two within six different methods in both the normal soil and the saline soil. The Roman numerals I, II, III, IV in the legend represented cellular processes, environmental information processing, genetic information processing, and metabolism of KEGG level one, respectively. The pathway of KEGG level one with a low percentage was just listed and not extended to KEGG level two (human diseases, organismal systems).
